# Type IV secretion systems: versatility and diversity in function

**DOI:** 10.1111/j.1462-5822.2010.01499.x

**Published:** 2010-09

**Authors:** Karin Wallden, Angel Rivera-Calzada, Gabriel Waksman

**Affiliations:** Institute of Structural and Molecular BiologyUCL and Birkbeck, Malet Street, London WC1E 7HX, UK

## Abstract

Type IV secretion systems (T4SSs) are large protein complexes which traverse the cell envelope of many bacteria. They contain a channel through which proteins or protein–DNA complexes can be translocated. This translocation is driven by a number of cytoplasmic ATPases which might energize large conformational changes in the translocation complex. The family of T4SSs is very versatile, shown by the great variety of functions among family members. Some T4SSs are used by pathogenic Gram-negative bacteria to translocate a wide variety of virulence factors into the host cell. Other T4SSs are utilized to mediate horizontal gene transfer, an event that greatly facilitates the adaptation to environmental changes and is the basis for the spread of antibiotic resistance among bacteria. Here we review the recent advances in the characterization of the architecture and mechanism of substrate transfer in a few representative T4SSs with a particular focus on their diversity of structure and function.

## Introduction

The type IV secretion system (T4SS) is one of several types of secretion systems, which microorganisms use for the transport of macromolecules such as proteins and DNA across the cell envelope (Rego *et al*., 2010). It is the most versatile family of secretion systems, mediating transport of monomeric proteins as well as multi-subunit protein toxins and nucleoprotein complexes, and has been found in both Gram-positive and Gram-negative bacteria as well as in some *archaea*.

There are broadly three functional types of T4SSs (Alvarez-Martinez and Christie, 2009). The first type, found in many Gram-positive and Gram-negative bacteria and some *archaea*, is used for transferring DNA from one cell to the other in a cell-to-cell contact-dependent manner. This event is called conjugation, and greatly increases genomic plasticity, helping microorganisms to adapt to changes in their environment. Conjugation is the major mechanism behind the spread of antibiotic resistance genes among pathogenic bacteria. Conjugative T4SSs are often encoded on self-transmissible plasmids together with genes that provide selective advantage for the cell such as antibiotic resistance, virulence traits or other metabolic functions that enhance survival. They can also be found as part of transposons integrated in chromosomes. Another T4SS transferring DNA is that of *Agrobacterium tumefaciens*, a well-characterized system specialized in delivering oncogenic nucleoprotein complexes into plant cells.

A second type of T4SSs mediates DNA uptake (transformation) and release from the extracellular milieu. The two so far characterized systems are the *Helicobacter pylori* ComB system, which take up DNA from the extracellular milieu, and the *Neisseria gonorrhoeae* gonococcal genetic island (GGI) which secretes DNA to the extracellular milieu.

A third type of T4SSs is used to transfer proteins. Most T4SSs in that category are found in pathogenic bacteria where they play important roles in virulence such as establishing pathogen–host interaction and/or transferring toxic effector proteins or protein complexes into the cytoplasm of the host cell. Important human pathogens like *H. pylori*, *Bordetella pertussis*, *Legionella pneumophila*, *Brucella* spp. and *Bartonella* spp. rely on T4SSs for their pathogenicity and the first three will be discussed later in more detail.

## Architecture of T4SSs

**Fig. 1:**
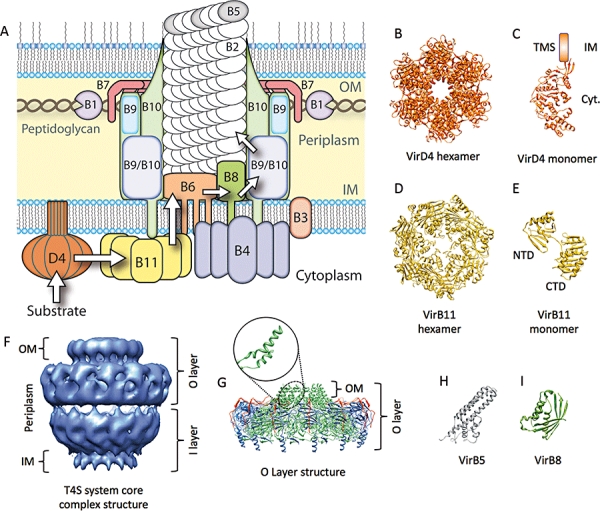
Schematic model of the VirB/D4 system of *A. tumefaciens* and structures of T4SS components determined to date. A. The experimentally predicted locations of VirB/D4 components of *A. tumefaciens*. Arrows indicate the sequential translocation steps identified during substrate secretion through the VirB/D4 system in *A. tumefaciens* (Cascales and Christie, 2004). B. Crystal structure of the TrwB hexamer, VirD4 homologue of the *E. coli* conjugative plasmid R388 (Gomis-Ruth *et al*., 2001). C. Single subunit of the TrwB hexamer with modelled plausible location of the N-terminal transmembrane domain. D. Crystal structure of the HP5025 hexamer, VirB11 homologue of *H. pylori* (Savvides *et al*., 2003). E. Single subunit of the HP0525 hexamer. The N-terminal (NTD) and C-terminal (CTD) domains are indicated. F. Cryo-electron microscopy structure of the T4SS core complex of the *E. coli* conjugative plasmid pKM101 (Fronzes *et al*., 2009b), comprising the full-length TraN, TraO and TraF, which correspond to the VirB7, VirB9 and VirB10 homologues of *A. tumefaciens* respectively. G. Crystal structure of the outer membrane complex, comprising the O-layer (Chandran *et al*., 2009). The inset shows the characteristic two-helix bundle of TraF that traverses the outer membrane. TraN, TraO and TraF are coloured red, blue and green respectively. H and I. Crystal structures of TraC, the VirB5 homologue encoded by pKM101 (Yeo *et al*., 2003), and the periplasmic C-terminal domain of VirB8 from *B. suis* respectively (Terradot *et al*., 2005). Colour-coding of subunits in (A) is preserved for the individual subunits shown in (B)–(E) and (G)–(I). OM, outer membrane; IM, inner membrane; TMS, transmembrane segment; Cyt., cytosol.

Details on architecture are only known for one particular class of T4SSs, termed ‘type IVA’, to which twoarchetypical T4SSs belong: that encoded by the Ti plasmid of *A. tumefaciens* and that encoded by the *Escherichia coli* pKM101 plasmid. The Ti plasmid system contains 12 proteins, VirB1–VirB11 and VirD4, which later in the review are referred to as the VirB/D4 proteins. Other type IVA systems have closely related homologues of most or all of these components. The structural knowledge of type IVA systems is summarized in Fig. 1.

The bulk of the proteins (VirB6–VirB10, and possibly VirB3) are likely to form the scaffold of the substrate translocation channel that spans the entire cell envelope (Fig. 1A). Structural studies have shown that VirB7, VirB9 and VirB10 assembles into a tightly associated ‘core complex’ containing 14 copies of each of the proteins (Chandran *et al*., 2009; Fronzes *et al*., 2009b) spanning the two membranes of Gram-negative bacteria. The structure of the core complex formed by the TraN, TraO and TraF proteins, the VirB7, VirB9 and VirB10 homologues, respectively, from the conjugative pKM101 T4SS, was determined at 15 Å using cryo-electron microscopy (Fronzes *et al*., 2009b). The core complex forms a cylindrical structure of 185 Å in diameter and 185 Å in length containing two layers termed O (outer) and I (inner) (Fig. 1F). A channel goes through the core complex connecting the cytosol to the extracellular milieu, with a cytoplasmic opening of 55 Å in diameter followed by a large chamber and then a small opening of 10 Å in diameter to the extracellular milieu. This 10 Å diameter channel is too small to let substrate out, suggesting that large structural changes must take place during substrate transfer.

Subsequently, the crystal structure of the entire O-layer (also termed the outer membrane complex) was determined at a resolution of 2.6 Å (Fig. 1G) (Chandran *et al*., 2009). This structure revealed that VirB10 forms the outer membrane channel. In fact, VirB10 is a very unusual protein since it inserts in both the inner and outer membranes (Chandran *et al*., 2009; Fronzes *et al*., 2009b; Jakubowski *et al*., 2009). Another remarkable feature of the outer membrane complex structure is that the outer membrane channel itself is formed by two-helix bundles contributed by 14 TraF/VirB10 proteins (Fig. 1G, inset). This α-helical barrel is a rare occurrence in outer membrane proteins. VirB10 is known to play an important role in the regulation of substrate transfer to the extracellular space. Because it spans the entire space between the membranes and is inserted in both, it is ideally positioned to play its regulatory roles, possibly sensing conformational changes due to substrate binding in the cytosol and relaying them to regulate outer membrane channel opening and closing. On the basis of observed stabilization interactions it was suggested that channel assembly is initiated by the assembly of the core complex between VirB7, VirB9 and VirB10 across the cell envelope. This complex forms spontaneously without energy requirements. Lipidation of VirB7 is needed for correct insertion of the core complex into the outer membrane and is essential for secretion (Fronzes *et al*., 2009a). The integral membrane proteins VirB6 and VirB8 are good candidates for forming the actual pore through the inner membrane of the T4SS, since they directly contact the substrate during secretion in *A. tumefaciens* (Cascales and Christie, 2004) (see below). Structures have been determined of VirB8 of *A. tumefaciens* (Bailey *et al*., 2006) and of the VirB8 homologue of *Brucella suis* (Terradot *et al*., 2005) (Fig. 1I).

VirB1 is a periplasmic lytic transglycosylase making holes in the peptidoglycan cell wall to allow the assembly of surface structures such as the pilus. In *A. tumefaciens*, it is cleaved into two parts, with the N-terminal part having the lytic transglycosylase activity (Baron *et al*., 1997). Both parts are required for wild-type virulence and the C-terminal region has been shown to be essential for T-pilus biogenesis (Llosa *et al*., 2000; Zupan *et al*., 2007).

VirB3 of *A. tumefaciens* is a small inner membrane protein that contains two transmembrane segments and is stabilized by VirB4 (Mossey *et al*., 2010), suggesting that they function together. This is supported by the occurrence of chimeric VirB3–VirB4 proteins, e.g. that of *Campylobacter jejuni*, and by the fact that the *virB3* gene most often is located directly upstream of *virB4* in T4SS operons.

VirB2 and VirB5 form pilus structures extending from the extracellular surface. The pilin VirB2 of *A. tumefaciens* forms the major pilus component and is essential for substrate transfer. VirB2 is cyclized via an intramolecular covalent head-to-tail peptide bond (Eisenbrandt *et al*., 1999). VirB5 of *A. tumefaciens* is a minor pilus component possibly functioning as an adhesin that, in some organism, localizes to the tip of the pilus (Aly and Baron, 2007; Kwok *et al*., 2007; Backert *et al*., 2008). The crystal structure of the VirB5 homologue TraC of the pKM101 plasmid has been determined (Yeo *et al*., 2003) (Fig. 1H). Many T4SS pili are similar to the P-pilus encoded by the RP4 (IncP) plasmid, such as those encoded by the R388 (IncW), pKM101 (IncN) and *A. tumefaciens* Ti plasmids. P-pili are 8–12 nm in diameter and less than 1 µm in length (Bradley, 1980; Lawley *et al*., 2003). The VirB2 pilin is thought to both stabilize a donor–host surface contact and to form a channel for the substrate transfer between the donor and host cells. The well-characterized F-pilus, encoded by the *E. coli* F-plasmid, has an 8–10 nm outer diameter, a length of 2–20 µm, and a channel within of 2 nm in diameter (Ippen-Ihler and Minkley, 1986; Lawley *et al*., 2003). The major F-pilus component is the TraA pilin (Lawley *et al*., 2003), which displays a weak homology to VirB2. DNA has been detected within the F-pilus (Shu *et al*., 2008) and DNA also appears to be transferred between cells that are not in direct contact during conjugation, possibly through the extended F-pilus (Babic *et al*., 2008).

Three ATPases, VirB4, VirB11 and VirD4, energize the system from the cytoplasm, driving the complex assembly and substrate translocation through the channel. Of the three T4SS ATPases, VirB4 is the one less characterized with no crystal structure solved to date. VirB4 proteins are attached to the inner membrane by one to three membrane-spanning segments or bind peripherally to the membrane through interactions with other T4SS components. Purified VirB4 proteins vary in oligomerization state depending on membrane association and solution conditions: monomeric, dimeric and hexameric forms have been reported (Dang *et al*., 1999; Rabel *et al*., 2003; Yuan *et al*., 2005; Arechaga *et al*., 2008; Durand *et al*., 2010). ATPase activity of VirB4 proteins has only been reported for hexameric forms (Arechaga *et al*., 2008; Durand *et al*., 2010), indicating that VirB4 functions as a hexamer, similarly to VirB11 and VirD4.

VirB11 belongs to a family of ATPases termed ‘traffic ATPases’, which are associated with Gram-negative bacterial type II, type III, type IV and type VI secretion systems (Fronzes *et al*., 2009a). VirB11-like ATPases are hexameric peripheral inner membrane proteins (Rashkova *et al*., 1997; Krause *et al*., 2000a), which display ATPase activity on their own (Rivas *et al*., 1997; Krause *et al*., 2000b). Crystal structures of the VirB11 homologues of the *H. pylori* Cag T4SS, HP0525 (Yeo *et al*., 2000; Savvides *et al*., 2003) (Fig. 1D and E) and of *B. suis* (Hare *et al*., 2006) have revealed the shape of a grapple, six N-terminal domains forming the base of the grapple and six C-terminal domains forming its claws (Fig. 1E).

VirD4 functions as a coupling protein (CP) in DNA-transferring systems, coupling DNA substrate processing and translocation, and as substrate receptor in effector translocation systems where it recruits substrates to the T4SS for secretion. The X-ray structure of the soluble, ∼50 kDa cytoplasmic domain of TrwB, the VirD4 homologue encoded by the *E. coli* IncW plasmid R388, revealed a globular hexameric assembly composed of two distinct domains with a ∼20-Å-wide channel in the middle (Gomis-Ruth *et al*., 2001) (Fig. 1B and C). VirD4-like proteins are attached to the inner membrane by their N-terminal membrane helix (Fig. 1C). This membrane anchor is required for oligomerization of TrwB (Moncalian *et al*., 1999; Hormaeche *et al*., 2002; Schroder *et al*., 2002) and could span the inner membrane providing a channel through which the DNA might pass by (Gomis-Ruth *et al*., 2001).

## General mechanism for substrate transfer

The mechanism of conjugation appears to be conserved in nearly all conjugation systems, independent of whether the system resides in a plasmid or is incorporated in a transposon. The mechanism of conjugation can be divided into three processes (Alvarez-Martinez and Christie, 2009). First, the DNA substrate is processed by proteins that assemble around the DNA at a conserved DNA sequence, the origin-of-transfer (OriT), forming a complex called the relaxosome. The relaxosome processing involves the nicking and unwinding of double-stranded (ds) DNA. The main player of the relaxosome is the relaxase which becomes covalently linked at the 5′ end of OriT. The DNA–relaxase complex is then recruited to the type IV secretion channel by ATPase homologues to the VirD4 CP component and is then translocated through the T4SS channel.

*Agrobacterium tumefaciens* utilizes a similar DNA processing mechanism as the conjugation systems, transporting a portion of the Ti plasmid DNA covalently linked to the relaxase VirD2. In *A. tumefaciens*, the DNA substrate interacts sequentially with several of the T4SS components (Cascales and Christie, 2004), starting with VirD4, which recruits the DNA substrate to the translocation channel (Atmakuri *et al*., 2003; Atmakuri *et al*., 2004). The substrate is then transferred to VirB11, independent of ATPase activity (Atmakuri *et al*., 2004), then to the inner membrane proteins VirB6 and VirB8, thereafter to VirB9 and finally to the pilus protein VirB2 (Fig. 1A). All three ATPases are vital for substrate transfer from VirB11 to the presumptive inner membrane channel complex. The exact role of VirB4 is yet unknown, although its importance is manifested by its evolutionary conservation throughout most, if not all, T4SS gene clusters known to date.

Protein effector translocation T4SSs rely on the general scaffolding described for conjugation systems to transport effector proteins directly to the cytosol of eukaryotic host cells in a cell-to-cell contact-dependent manner. They rely with some exceptions on ATPases homologues to VirD4 for recruiting the effector substrate to the T4SS channel.

Regarding T4SSs involved in DNA uptake, recent data on the *H. pylori* ComB T4SS indicate that this system is part of a two-step DNA-uptake mechanism. In the suggested mechanism, the ComB proteins, encoded on two operons *comB2-B4* and *comB6-B10*, mediate transport of dsDNA across the outer membrane, and the inner membrane channel, ComEC, encoded on a separate operon, mediate import of single stranded DNA to the cytoplasm (Stingl *et al*., 2010).

## Classification of the T4SSs

**Fig. 2:**
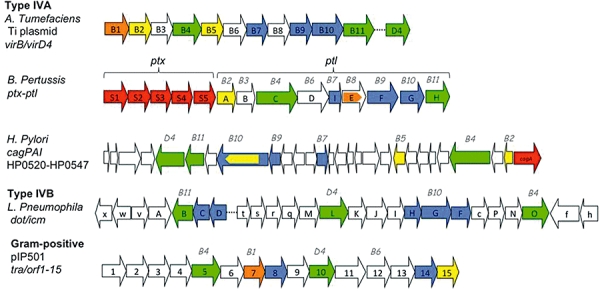
Genetic organization of T4SSs discussed in detail in this review. Homology to the *virB/D4* system is indicated by the text in grey above the schemes, e.g. *B4* above a gene means that this gene is homologous to *virB4*. Genes in blue corresponds to plausible core components, in green ATPases, in yellow plausible surface components, in orange lytic tranglycosylases and in red effector proteins. For *L. pneumophila*, upper-case gene names = Dot and lower-case gene names = Icm.

Type IV secretion systems can be divided into subgroups based on gene structure and evolution (Fig. 2). VirB4 is conserved in all characterized T4SSs and can be used as a signature for classification purposes. A few different classification schemes for T4SSs have been proposed from phylogenetic analysis or similarities to prototypical conjugative plasmids. The phylogenetic classifications are mostly based on the analysis of genes homologous to *virB10*, *virB4* and *virD4* of *A. tumefaciens*. VirB10 has not been found in Gram-positive bacteria and *archaea* (see below) and can therefore only help in defining T4SSs of Gram-negative bacteria. VirD4 is found in most T4SSs but is less suitable than VirB4 because of its high sequence variability (Juhas *et al*., 2008).

Initially, T4SSs were subgrouped into types F, P and I based on similarity to the conjugative plasmids IncF (plasmid F), IncP (plasmid RP4) and IncI (plasmid R64) respectively (Lawley *et al*., 2003). Subsequently, an alternative classification scheme based on similarities to the *A. tumefaciens* VirB/D4 system differentiates between type IVA (type F and P) and type IVB (type I) (Christie *et al*., 2005). Type IVA systems resemble the prototypic VirB/D4 system of *A. tumefaciens*, whereas type IVB system proteins are only distantly related to the VirB/D4 proteins. The most characterized type IVB system is the Dot/Icm (defective in organelle trafficking/intracellular multiplication) systems identified in the intracellular bacterial pathogens, *L. pneumophila* and *Coxiella burnetii*.

The emerge of antibiotic resistant strains of Gram-positive bacteria that cause hospital-acquired diseases such as the methicillin-resistant *Staphylococcus aureus* (MRSA) has led to the identification of broad-host-range plasmids that carry antibiotic resistance and are conjugated through T4SSs. One such plasmid that encodes a T4SS is the broad-host-range pIP501 plasmid that will be discussed later (Fig. 2).

Finally, some conjugative T4SSs found in transposons have been categorized into the sublineages of the ICE*Hin*1056 system of *Haemophilus influenzae*, the pKLC102/PAPI systems of *Pseudomonas aeruginosa* and the SPI-7 system of *Salmonella enterica typhi* (Juhas *et al*., 2008). These sublineages are only distantly related to the *A. tumefaciens* VirB/D4 system.

## Diversity/similarity of T4SSs at a molecular level

Because of the versatility of the T4SS, it is not difficult to imagine a rather large diversity of protein composition among systems. Naturally a subset of VirB/D4 homologues is present in all systems, since they have a common evolutionary origin, but this subset of components can vary in both number and identity. Below we review a few examples of this.

A well-studied type IVA system of a human pathogen is that encoded on the *cag* (cytotoxin-associated genes) pathogenicity island (*cag*PAI) found in many strains of *H. pylori*. This pathogen lives near the surface of the human gastric mucosa and is the main causing agent of peptic ulcer disease, mucosa-associated lymphoid tissue lymphoma and gastric adenocarcinoma (Backert and Meyer, 2006). The *cag*PAI encodes proteins homologous to VirB2, VirB4, VirB5, VirB7, VirB9, VirB10, VirB11 and VirD4, and a group of proteins with no sequence similarity to *A. tumefaciens* VirB/D4 proteins (Fig. 2).

The *cag*PAI mediates formation of pilus structures of 100–200 nm in length (Rohde *et al*., 2003; Kwok *et al*., 2007). The VirB2 homologue HP0546 was identified as the major pilus component (Andrzejewska *et al*., 2006). CagL (HP0539), which shows some sequence similarity to VirB5, was shown to mediate host cell recognition or adhesion (Kwok *et al*., 2007). The pilus base is associated with VirB7 (CagT; HP0532) and VirB9 (CagW; HP0528) homologues (Rohde *et al*., 2003; Tanaka *et al*., 2003). Interestingly, the VirB10 homologue HP0527 also forms an extended surface appendage (Rohde *et al*., 2003), which could be a unique feature of the *cag* T4SS. HP0527 varies in size among strains but is unusually large (up to 2000 residues) compared with other VirB10 homologues found in databases, which commonly contain between 300 and 600 residues. The most conserved region between VirB10 proteins correspond to the approximately 200 residues forming the outer membrane channel of the pKM101 system (Fig. 1G) (Chandran *et al*., 2009). This conserved region is at the very C-terminus of HP0527. The remaining part of the protein contains, besides a probable inner membrane-spanning region, a large number of repeat regions including regularly distributed cysteins, which are likely to help forming a rigid surface structure. The repeat regions can be deleted or extended by intragenic recombination to enable variation between strains, helping this pathogen to avoid host antibody recognition (Rohde *et al*., 2003). A question that remains to be answered is how HP0527 can both have a central role in substrate translocation and be a part of surface filaments. One possibility could be that the protein is processed during the assembly of the complex, leaving the conserved VirB10 region and the inner membrane region as part of the pore and exporting the bulk of the protein to form a surface filament.

The *cag*PAI also encodes the effector protein CagA, which is thought to be the major virulence factor of *H. pylori*, mainly since CagA-positive but not CagA-negative *H. pylori* strains are associated with the development of severe gastric diseases (Backert and Meyer, 2006). CagA has been visualized at the tip of the pilus (Rohde *et al*., 2003; Kwok *et al*., 2007) and is thought to be ‘injected’ through the pilus directly into the cytosol of the host cell. However functional activity of the T4SS pilus requires integrin receptors of the host (Backert and Selbach, 2008), which are recognized by the CagL protein (Kwok *et al*., 2007).

Another T4SS is found in *B. pertussis*, the causative agent of a severe respiratory infection known as whooping cough. The genes encoding this system are clustered on a single operon called *ptl* (pertussis toxin liberation) (Weiss *et al*., 1993) (Fig. 2). No homologues have been found for *virB1*, *virB5* and *virD4*. Lack of VirB1 could be partially compensated by PtlE, the VirB8 homologue in *B. pertussis* T4SS, since it exhibits peptidoglycanase activity (Rambow-Larsen and Weiss, 2002). On the other hand the absence of a VirD4 homologue could be the consequence of a different effector translocation mechanism as discussed below. *B. pertussis* T4SS functions by secreting its substrate into the extracellular milieu instead of injecting it across host cell membranes. No pilus structure has been found associated with the Ptl system, although substrate secretion depends on PtlA, the VirB2 pilin homologue (Craig-Mylius and Weiss, 1999).

The effector proteins secreted by the Ptl machinery form a large complex called the pertussis toxin (Ptx). The corresponding *ptx* genes are located directly upstream of the *ptl* genes, and the *ptx* and *ptl* genes are co-transcribed (Fig. 2) (Burns, 2003). Ptx is an AB exotoxin which is composed of five different protein subunits (S1–S5) that are translocated into the periplasm where the assembly of the holotoxin takes place. Distinctly to most of the known effectors from other T4SSs, the Ptx subunits have their own signal sequences; so they are translocated across the inner membrane by the general SecYEG secretory pathway (Nicosia *et al*., 1986). Although extremely intriguing, the mechanism by which this holotoxin with an overall dimensions of 6 nm × 6 nm × 10 nm is translocated across the T4SS remains obscure. It has been proposed that the assembly of a functional Ptl secretion system depends on interactions with the Ptx toxin in the periplasm, and only after this interaction has taken place the Ptx can be secreted to the extracellular milieu (Verma and Burns, 2007). Once secreted Ptx binds to sialic acids of different host cell glycoproteins and is endocytosed.

*Legionella pneumophila* is a Gram-negative facultative intracellular parasite, which is the causative agent of a type of pneumonia called Legionnaire's disease. It has a history of contaminating water distribution systems and can be fatal for patients with compromised immune systems. Normally *L. pneumophila* uses amoebae and protozoa as a reservoir, but it can also be present in contaminated aerosols and, when inhaled, can infect alveolar macrophages. Importantly, after being phagocytosed *L. pneumophila* is able to avoid subsequent lysis thanks to its ability to subvert normal phagosome maturation. The *L. pneumophila* Dot/Icm T4SS is involved in the inhibition of the phagosome–lysosome fusion and in facilitating the recruitment to the rough ER in order to favour bacterial replication inside the host cell. Numerous effector proteins are translocated by the Dot/Icm system of *L. pneumophila* that enables the survival within the phagosomes (Ensminger and Isberg, 2009).

Twenty-five *dot*/*icm* genes have been identified in two regions of the chromosome of *L. pneumophila* (Fig. 2), which are essential for the outcome of the infection and for achieving intracellular survival (Juhas *et al*., 2008). Five Dot/Icm proteins (DotC, DotD, DotF, DotG and DotH) appear to form a subcomplex that bridges the inner and outer membranes and are likely to exhibit analogous function as the type IVA VirB7–VirB10 complex. DotC and DotD are both periplasmic lipoproteins. DotH is tightly associates with DotC and DotD (Vincent *et al*., 2006). DotG, which is a large protein of 1048 amino acids, shows weak homology to VirB10 at its C-terminus, indicating that it might traverse the outer membrane similarly to type IVA VirB10 proteins (Fig. 1).

Five Dot/Icm proteins (DotB, IcmQ, IcmR, IcmS and IcmW) are cytoplasmic, of which the hexameric ATPase, DotB, is homologous to VirB11 and plays a role in the selection of substrates for secretion (Sexton *et al*., 2005). DotL and DotO show weak sequence identity to the ATPases VirD4 and VirB4 respectively.

The *L. pneumophila* Dot/Icm system appears to lack a pilus, which is consistent with the system functioning within the host cell. This system translocates more than 50 effectors during the infectious process (Ninio and Roy, 2007), including RalF, a guanine exchange factor that activates ADP-ribosylation factor 1, DotA and a number of proteins called Sids (substrates of Icm/Dot transporter).

Antibiotic resistance genes found in a variety of broad-host-range plasmids/transposons, are spreading in a range of bacteria in health-care settings. To date the most characterized T4SSs involved in this are found in plasmids of Gram-positive bacteria, e.g. enterococci, streptococci and staphylococci, which are the most common causes of hospital-acquired infections (Grohmann *et al*., 2003). Recently, new strains of MRSA have been discovered causing also community-acquired infection in patients without previous healthcare contacts (Otter and French, 2010).

The plasmid pIP501 has the broadest known host range for a conjugative plasmid originating from Gram-positive bacteria. It can self-transfer to a variety of Gram-positive bacteria such as streptococci, staphylococci, enterococci, listeria, multicellular *Streptomyces lividans*, and also to the Gram-negative *E. coli* (Kurenbach *et al*., 2003).

The pIP501 conjugation system is encoded on a single operon of 15 *tra* genes (Fig. 2). Components homologous to VirB1, VirB4, VirB6, VirD4 and the relaxase VirD2 have been found, being so far named Orf7, Orf5, Orf12, Orf10 and Orf1 respectively. These components have been found in most Gram-positive conjugative plasmids/transposons including the broad-host-range pAM1, pRE25 and Tn*916* of *Enterococcus faecalis* and Tn*1545* of *Streptococcus pneumoniae*, and the more host-specific pSK41 and pGO1 of *S. aureus* and pCF10 of *E. faecalis*. Homologues of VirB11 have been found in pXO1 and pXO2 of *Bacillus anthracis* (Grohmann *et al*., 2003).

The pIP501 *tra* operon is negatively autoregulated at the transcriptional level by the first gene product of the operon, the TraA relaxase (Orf1). The TraA protein has a similar role as the archetypical VirD2 protein of *A. tumefaciens*, reacting covalently to the 5′ end of the DNA substrate.

All known Gram-positive T4SSs encode a VirB4 homologue and many of them encode a small protein with two or three predicted *trans*-membrane regions directly upstream of VirB4, e.g. PrgI which is directly upstream of the VirB4 homologue, PrgJ, in pCF10. It was postulated that these two gene products function together (Alvarez-Martinez and Christie, 2009). In pIP501, this transmembrane protein is represented by Orf3, which is homologous to PrgI and shows some similarity to the VirB3 protein of type IVA systems. No interaction has been established between Orf3 and the VirB4 homologue Orf5. Instead Orf5 interacts with the VirB1 homologue Orf7, and two uncharacterized components Orf4 and Orf14 (Abajy *et al*., 2007).

Gram-positive T4SSs appear to have a weak homologue of the lytic tranglycosylase VirB1, but of approximately twice the size, e.g. Orf7 of pIP501 is 40.5 kDa compared with the < 25 kDa Gram-negative homologues. In Gram-negative bacteria, this protein digests the periplasmic peptidoglycan layer enabling the assembly of the outer membrane structure of the complex. In Gram-positive bacteria, it instead probably digests the much thicker multilayered structure of peptidoglycan found on the outer surface.

Since Gram-positive bacteria and *archaea* lack an outer membrane, it is not a surprise that homologues of VirB7, VirB9 and VirB10 (forming the outer membrane complex of IVA systems) have not been found in neither Gram-positive bacteria nor *archaea*. Instead in pIP501, Orf8 and Orf14 were suggested to constitute an important part of the translocation complex. The weak VirB6 homologue, Orf12, which interacts with Orf14 could also have an important structural role (Abajy *et al*., 2007). A component of pIP501 that might be suggested to be involved in surface structures is Orf15. It is homologous to PrgC of pCF10, carries a C-terminal cell wall anchor-like motif and contains 47 tandem repeats of prolins and glutamates/aspartates which are common in surface structures of Gram-positive bacteria (Alvarez-Martinez and Christie, 2009).

## Conclusion

The T4SS has evolved to deal with many different tasks from conjugation to transport of sometimes more than 50 different effector proteins through the same system, as shown by the *L. pneumophila* Dot/Icm T4SS. It is also present in a very large number of organisms from Gram-positive and Gram-negative bacteria to *archaea*. It is however absent in eukaryotes although it cannot be excluded that weak sequence homology has prevented their characterization in these higher organisms. In a number of cases, T4SSs have evolved to be part of the formidable arsenal of pathogenic bacteria.

As this review attempts to demonstrate, such versatility is likely to be reflected in the system's architecture. This is becoming more and more apparent with the increasing number of T4SS homologues being identified as a result of genome sequencing: indeed, they demonstrate a previously unsuspected variation in sequence and composition that is bound to affect the overall structure of the system. In this respect, the functional and structural characterization of T4SSs other than the one encoded by the pKM101 system (the most thoroughly studied in structural terms) is going to be crucial in order to understand the secretion process of this machinery and, more importantly, to understand its implications in the infectious mechanism of critical human pathogens.
